# Preliminary analysis of immune activation in early onset type 2 diabetes

**DOI:** 10.3402/ijch.v72i0.21190

**Published:** 2013-08-05

**Authors:** Julia D. Rempel, Juliet Packiasamy, Heather J. Dean, Jonathon McGavock, Alyssa Janke, Mark Collister, Brandy Wicklow, Elizabeth A. C. Sellers

**Affiliations:** 1 OOH-QUIN Immunology Laboratory, Section of Hepatology, Department of Internal Medicine, Manitoba Institute of Child Health, Winnipeg, Canada; 2 Department of Immunology, University of Manitoba, Winnipeg, Canada; 3 Manitoba Institute for Child Health, University of Manitoba, Winnipeg, Canada; 4 Department of Pediatrics, University of Manitoba, Winnipeg, Canada

**Keywords:** early onset type 2 diabetes, TLR4, interleukin 1beta

## Abstract

**Introduction:**

First Nations and other Aboriginal children are disproportionately affected by cardiometabolic diseases, including type 2 diabetes (T2D). In T2D, the disruption of insulin signalling can be driven by pro-inflammatory immunity. Pro-inflammatory responses can be fueled by toll-like receptors (TLR) on immune cells such as peripheral blood mononuclear cells (PBMC, a white blood cell population). TLR4 can bind to lipids from bacteria and food sources activating PBMC to produce cytokines tumour necrosis factor (TNF)-α and interleukin (IL)-1β. These cytokines can interfere with insulin signalling. Here, we seek to understand how TLR4 activation may be involved in early onset T2D. We hypothesized that immune cells from youth with T2D (n=8) would be more reactive upon TLR4 stimulation relative to cells from age and body mass index (BMI)-matched controls without T2D (n=8).

**Methods:**

Serum samples were assayed for adipokines (adiponectin and leptin), as well as cytokines. Freshly isolated PBMC were examined for immune reactivity upon culture with TLR4 ligands bacterial lipopolysaccharide (LPS, 2 and 0.2 ng/ml) and the fatty acid palmitate (200 µM). Culture supernatants were evaluated for the amount of TNF-α and IL-1β produced by PBMC.

**Results:**

Youth with T2D displayed lower median serum adiponectin levels compared to controls (395 vs. 904 ng/ml, p<0.05). PBMC isolated from youth with and without T2D produced similar levels of TNF-α and IL-1β after exposure to the higher LPS concentration. However, at the low LPS dose the T2D cohort exhibited enhanced IL-1β synthesis relative to the control cohort. Additionally, exposure to palmitate resulted in greater IL-1β synthesis in PBMCs isolated from youth with T2D versus controls (p<0.05). These differences in cytokine production corresponded to greater monocyte activation in the T2D cohort.

**Conclusion:**

These preliminary results suggest that cellular immune responses are exaggerated in T2D, particularly with respect to IL-1β activity. These studies aim to improve the understanding of the biology behind early onset T2D and its vascular complications that burden First Nations people.

Metabolic syndrome (MetS) and type 2 diabetes (T2D) present a significant burden to Canadian First Nations and other Indigenous populations (1). More troubling is that these metabolic diseases, which were once restricted to adults, are becoming increasingly prevalent in children and youth (2). Within Canada, Manitoba has the highest incidence of early onset T2D, with First Nations being disproportionately affected (3,4).

The increasing prevalence of T2D among Indigenous youth worldwide can be attributed to both genetic and environmental factors (5,6). Significant environmental changes include a shift away from traditional food to nutrient sparse, calorie dense, westernized food, as well as an increasing sedentary lifestyle. The shift away from a more traditional lifestyle is reflected in the considerable rates of obesity within First Nations youth in Canada (7,8). Obesity is a significant determinant of MetS and T2D (9, 10). In one First Nations community, obese children had a 5.1 odds ratio (95% CI 1.51, 17.0) of developing T2D before the age of 18 years (11).

## Adipose tissue as immune tissue in T2D

The immune system is a critical mediator in the onset of T2D. Adipose tissue is not inert, but acts as inflammatory immune tissue. Adipose tissue consists of adipocytes that secrete adipokines or “fat hormones” such as apidonectin and leptin. These adipose-derived hormones influence insulin sensitivity and therefore play a role in maintaining normal glucose levels. Adiponectin levels are decreased in states of metabolic disease; whereas, leptin concentrations are often increased (12, 13). However, the role of adipokines in the natural history of early onset T2D is poorly understood.

## Toll-like receptor 4 and sterile inflammation

Adipose tissue also contains macrophages, which can account for more than 25% of cells within the adipose tissue (14). When macrophages move into the blood stream they take on different characteristics and are called monocytes. Macrophage/monocytes interact with lipids through receptors on their surface including toll-like receptor (TLR)4. TLR4 is important in protecting the body from bacterial infections through binding lipopolysaccharides (LPS) found on the surface of Gram-negative bacteria such as *E. coli*. The binding of TLR4 to LPS in a bacterial infection results in the macrophages/monocytes becoming activated allowing them to produce pro-inflammatory cytokines, which assist in clearing the infections. However, chronic exposure to these cytokines can be harmful.

Chronic cytokine exposure can occur upon consumption of diets high in lipids. These lipid complexes can also bind TLR4 receptors causing cells to become activated. Palmitate is a fatty acid that is present in many foods. Increased serum palmitate levels are associated with a high degree of liver steatosis (15). In addition, palmitate exposure can increase TLR4 levels on macrophage cell lines 8-fold, as well as activate TLR4 resulting in pro-inflammatory cytokine production (16). Similar findings were observed with human monocytes (16).

## Cytokines in T2D

Many cytokines have been implicated in obesity-induced inflammation and T2D. Our focus has been on tumour necrosis factor (TNF)-α, interleukin (IL)-1β and IL-6. These cytokines can impair insulin signalling or induce β-cell apoptosis (17–19). However, it is only in cases of extreme immune activation that cytokine spillage into the blood stream occurs. Thus, examination of TLR4 responsiveness requires an assessment of cellular activity.

## Immunity in Manitoban Indigenous populations

TLR4 activation can upset the normal balance of the immune system promoting insulin resistance. This can lead to an increased risk for cardiovascular and other diseases (20–22). The relevance of TLR4-induced cytokine activity in early onset diabetes in Aboriginal peoples is unknown. Serological studies, examining immune markers in the serum have had limited findings (23). However, previous studies by our unit and others indicated a marked difference in immune genetics between Manitoban Indigenous peoples and Caucasians (5,6, 24). An assessment of cellular immune function, examining peripheral blood mononuclear cell (PBMC) cytokine production, also indicated that First Nations adults have greater inflammatory responses than Caucasians (5).

The heightened pro-inflammatory immunity observed in these studies, concomitant with stressful environments and changes in lifestyle, could promote an earlier onset of T2D. Altogether, these factors could enhance *susceptibility* to, and *progression* of, T2D, as well as promote the high prevalence of T2D-related co-morbidities observed in this young population (25–27).

## Study goal

The purpose of this study is to evaluate systemic (evaluated in serum) and cellular (examined with PBMC) immunity in youth with T2D relative to age- and body mass index (BMI)-matched normoglycemic youth to determine the role of the immune system in early onset T2D. We hypothesized that immune cells from youth with T2D would be more reactive upon TLR4 stimulation compared to PBMC from youth without T2D.

## Methods

### Subjects

This study was approved by the University of Manitoba Research Ethics Board and Health Sciences Centre (HSC) Research Board, Winnipeg, Manitoba. In addition to ethical approvals, progress reports were presented to the Manitoba First Nation Diabetes Committee, an advisory committee of individuals who work in Manitoban First Nations communities, which is funded by the diabetes programme of the First Nations and Inuit Health Branch of Health Canada. Youth with T2D were recruited through the paediatric endocrinology clinic, Winnipeg Children's Hospital, Winnipeg, MB. Overweight youth without T2D were recruited through the Manitoba Institute of Child Health, a research unit serving a large geographic region of central Canada. Youth (14–18 yrs old) qualifying for the study were approached by a clinical research coordinator. Written informed consent was given by parents or guardians. Participants provided a signature of assent to state agreement to their involvement. A short questionnaire inquiring about general health, co-morbidities and medications was also administered. Ethnicity was self-declared as First Nations or Métis. All other groups were designated as non-Aboriginal.

Individuals with chronic infections, chronic inflammatory disease and/or signs of acute infection (cold, flu, malaise) were not recruited. We also excluded individuals with the known hepatic nuclear factor (HNF)-1α G319S polymorphism (GS or SS genotype). The HNF-1α G319S polymorphism is a private polymorphism associated with T2D in the Oji-Cree First Nations population in Manitoba and northwestern Ontario. It results in a mild insulin secretory defect and is associated with early onset T2D in this population (28,29).

### Clinical parameters

Participants were weighed in kilograms using a standard office scale. Height (in centimetres) was assessed using a stadiometer. BMI was computed from height and weight (height/m^2^). Obesity was defined as≥95 percentile for age and gender (30). Blood pressure was measured in the sitting position using a standard sphygmomanometer. Clinical chemistry was determined at the Clinical Chemistry Department, HSC.

### Blood sample collection and PBMC isolation

Serum samples and whole blood were collected in the morning. Serum samples were stored at −80°C until analysis of cytokines by ELISA. ELISAs were performed as previously described (31). Adiponectin and leptin ELISAs were purchased from R&D Systems (Minneapolis, MN, USA).

PBMC are a white blood subset containing monocytes and lymphocytic cells including T cells and B cells. PBMC were isolated from whole blood with Ficoll (Sigma, St. Louis, MO, USA) as previous described (5,31). Cells consistently exhibited>98% viability (5,31).

### In vitro culture and cytokine protein analysis

Freshly isolated PBMC were cultured at 0.25×10^6^ cells/ml in 96-well round bottom plates (Corning Inc., Corning, NY, USA) and incubated with culture medium, TLR4 ligands LPS (2 and 0.2 ng/ml, Sigma) or palmitate (200 µM, Sigma) conjugated to bovine serum albumin. Palmitate was conjugated as previously described (16). Supernatants were harvested 24 hours later for the detection of cytokine levels.

### Intracellular cytokine staining

Briefly, freshly isolated PBMC were cultured (0.25×10^6^ cells/well) in the presence of medium, LPS (20 ng/ml) or palmitate (200 µM) along with Brefeldine A (10 µg/ml, BD Biosciences) for 4 hours. Brefeldine A inhibits secretion of protein from cells. At 4 hours, cells were washed. Fluorochrome-conjugated anti-CD14, an antibody that detects monocytes, was added for 30 minutes at 4°C. Cells were washed with 0.01% saponin solution to permeablize the cells so that the antibodies could penetrate the cell membrane. Fluorochrome-conjugated antibodies for intracellular staining against TNF-α and IL-1β were added for 30 minutes in the dark. Cells were washed and stored at 4°C in the dark. The next day, the data were acquired on a BD FACSCanto II flow cytometer. This machine allows visualization of the fluorochromes so that the percentage of cells bound by corresponding antibodies can be assessed.

### Data analysis

Categorical differences were determined by χ^2^ Fisher's exact test. The Mann-Whitney test was used to determine if significant differences existed between the presence and absence of T2D. Spearman's correlation was used to determine relationships between immune and clinical parameters. p<0.05 was considered significant.

## Results

### Patient demographics

This preliminary report details findings from youth with (n=8) and without (n=8) T2D. Demographic profiles were similar, except that the T2D cohort contained a greater percentage of First Nations youth relative to the non-T2D cohort (p<0.05, Table I). Youth with T2D also displayed slightly higher resting systolic blood pressure (p<0.05).

**Table I:** Study cohorts

Parameters	T2D^b^ (n=8)	Control (n=8)
Demographics		
Age (years)^a^	15 (15–17)	16 (14–17)
Female (%)	87.7	75
First Nation (%)	87.5	25*
Métis (%)	0	25
Non-Aboriginal (%)	12.5	50
Clinical and laboratory		
BMI^a^	27 (22.2–42.2)	29 (25.1–38.7)
Blood pressure^a^		
Systolic (mmHg)^a^	129 (105–139)	106 (102–136)*
Diastolic (mmHg)^a^	67 (57–84)	64 (53–74)
Triglyceride (mmol/L)^a^	1.6 (0.6–3.5)	2.0 (1–3.4)
ALT (IU/l)^a^	20.0 (10–46)	14.0 (11–46)
AST (IU/l)^a^	21.5 (11–31)	20.0 (13–33)

a Median and range shown.

b Quantitative data were assessed by Mann–Whitney. Categorical differences were determined by χ^2^ Fisher's exact test.

* p<0.05 was considered significant. BMI, body mass index; ALT, alanine aminotransferase; AST, aspartate aminotransferase.

### Adiponectin levels were lower in early onset T2D

**Fig. 1:**
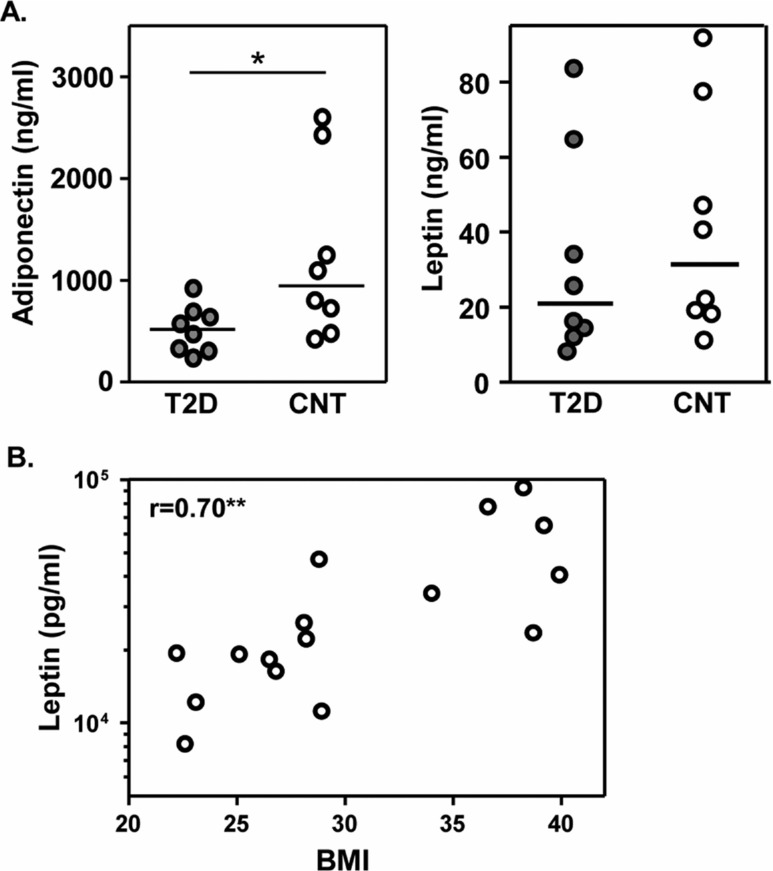
Adipokine associations with disease parameters. Serum adiponectin and leptin concentrations were assessed by ELISA. A. Adiponectin levels were significantly lower in T2D youth, compared to obese matched controls. Horizontal bars indicate median values (Mann-Whitney, *p<0.05). B. Leptin concentrations correlated with BMI. Relationships were assessed by Spearman correlation (**p<0.01). Serum samples were also analyzed for cytokines TNF-a and IL-6 based on previous studies by group members. However, serum cytokines were undetectable in these subjects (data not shown).

Adiponectin concentrations were lower in the T2D cohort relative to the control cohort (p<0.01, Fig. 1); whereas leptin levels were similar between the groups. Leptin levels, however, positively correlated with BMI (p<0.01). Circulating TNF-α and IL-6 levels, an indication of systemic immune activation, were undetectable in these youth cohorts.

### Cellular immune sensitization to TLR4 activation in youth with and without T2D

**Fig. 2:**
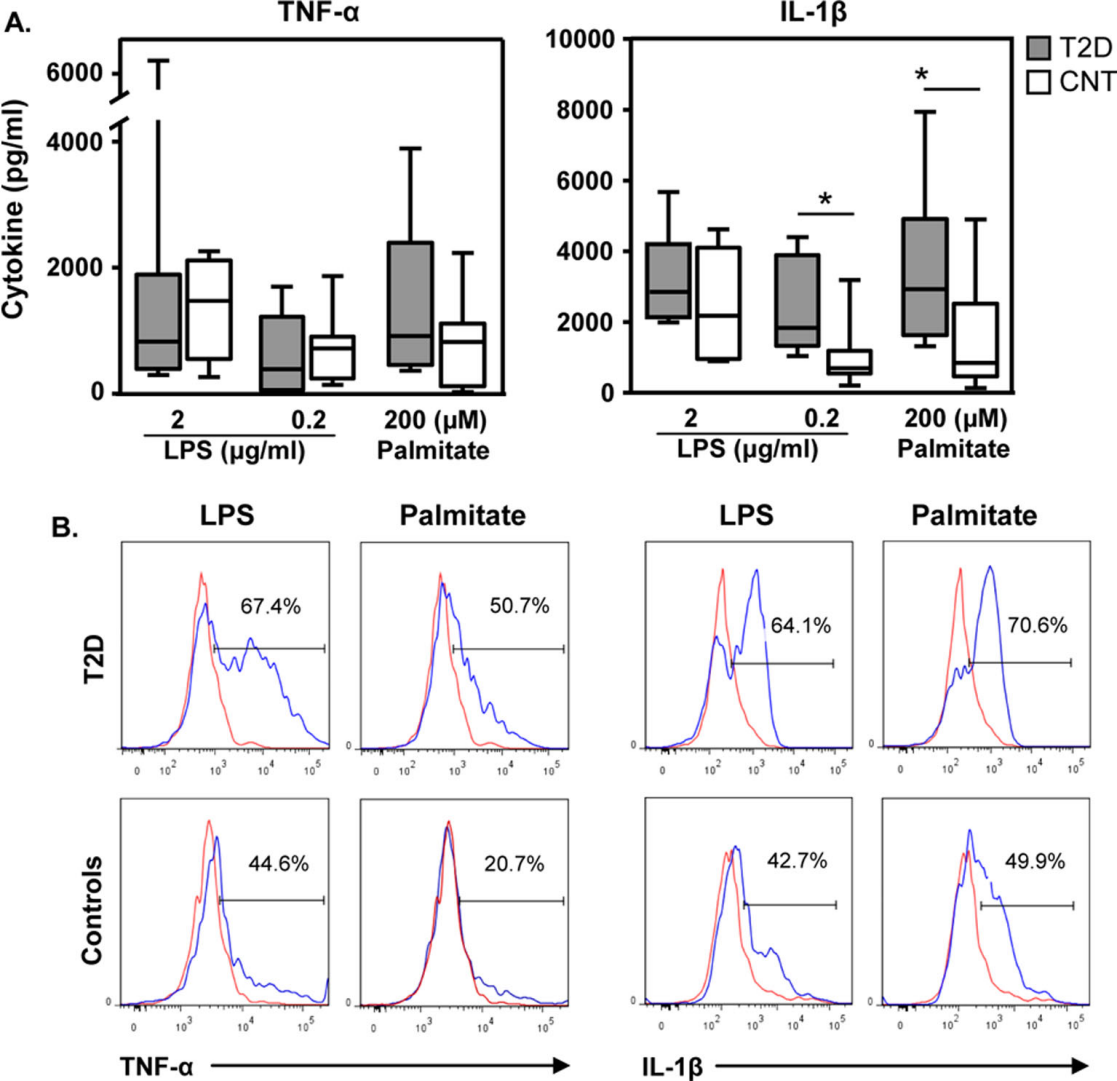
T2D cohort demonstrates enhanced cellular sensitivity to TLR4 ligands than obese controls. A. PBMC from youth with (n=8, grey bars) and without (n=8, white bars) T2D were cultured as described in Methods. Whisker plots show medians and ranges (Mann Whitney, *p<0.05). B. PBMC from youth with (n=3) and without T2D (n=3) were activated for 4 hrs with LPS and palmitate. Cells were stained as per Methods (ICCS). Shown are PBMC gated on the CD14 positive cells, the monocyte population. Red lines indicate responses to culture medium alone. Blue lines indicate responses to either LPS or palmitate as indicated. One set of 3 representative results is shown.

To assess whether sensitivity to TLR4 activation in T2D and obese youth cohorts differed, PBMC were incubated with culture medium, LPS (2 or 0.2 µg/ml) or palmitate. Independent of LPS or palmitate stimulation TNF-α production was comparable between T2D and control cohorts (Fig. 2A). IL-1β secretion was also similar between cohorts at the high LPS concentrations. In contrast, at the low LPS dose PBMC from the T2D cohort were more reactive for IL-1β synthesis than cells acquired from obese youth without T2D (medians, 1,745 vs. 705 pg/ml, p<0.05). Moreover, following palmitate activation PBMC secretion of IL-1β was 3.5-fold greater from the T2D cohort relative to their counterparts (medians, 2,927 vs. 849 pg/ml, p<0.05). TNF-α and IL-1β synthesis did not correlate with clinical parameters (data not shown).

PBMC consist of monocyte and lymphocyte populations. Because of their putative role in T2D, monocyte reactivity to LPS and palmitate was directly examined with intracellular cytokine staining. A greater percentage of monocytes from the T2D cohort (n=3) were actively producing TNF-α and IL-1β in response to TLR4 activation by LPS than from monocytes from the control cohort (n=3, Fig. 2B). Palmitate exposure for 4 hours also resulted in a greater percentage of monocytes from youth with T2D than youth without T2D being involved in TNF-α and IL-1β synthesis.

## Discussion

Inflammatory immunity stemming from adipose tissue is a critical factor in the onset and progression of T2D in adult human and animal models. The primary novel finding from this preliminary cross sectional study of immune reactivity in early onset T2D was that the immune response from youth with T2D was hyperreactive to long chain fatty acids compared to obese matched youth without T2D.

### Adipokines

Adiponectin levels are lower among overweight individuals with metabolic diseases, in particular dysglycemia (32–35). The data presented here extend these findings by demonstrating that adiponectin concentrations were also lower in youth with T2D compared to normoglycemic controls (Fig. 1A). Studies in Oji-Cree populations revealed that adiponectin levels are prognostic for (23,36). Whether hypoadiponectinemia is a cause or consequence of dysglycermia in youth has yet to be determined. Prospective cohort studies of obese youth are needed to determine its role in the natural history of early onset T2D.

### Systemic immunity

Systemic immunity reflects the background inflammatory status of the body, representing the “spill over” from cellular events. In adults, serum pro-inflammatory cytokines such as TNF-α and IL-6 are elevated in those with obesity and T2D relative to healthy controls (37). Here, serum TNF-α and IL-6 were undetectable. The absence of serum cytokines, a common finding in obese adults suggests that the duration of T2D affects the extent of systemic inflammation. In a study of 362 children, low serum TNF-α levels did not correlate with metabolic syndrome or BMI (38). However, associations of pro-inflammatory cytokines with obesity in adolescents have been observed (33). Stringer et al. also found that serum IL-6, but not TNF-α, levels were higher in T2D (n=24) relative to obese matched (n=19) First Nations youth (23). The difference between the results of these studies is unclear. Both studies have a small sample size and different individual subjects.

### Cellular immunity

In addition, the susceptibility of PBMC to TLR4 activation was examined by culturing freshly isolated PBMC with LPS and palmitate. LPS- or palmitate-induced TNF-α did not differentiate with T2D diagnosis (Fig. 2A). Similar results were observed for IL-1β production upon activation with the higher LPS dose. However, at the low LPS dose (0.2 µg/ml), the cells derived from the T2D cohort secreted 2.3-fold more IL-1β than their counterparts (p<0.05). Thus, in early onset T2D, peripheral immune cells appear to have a lower threshold for LPS-induced IL-1β synthesis. Moreover, palmitate activation induced higher median levels of IL-1β from the T2D cohort versus the obese control cohort (2,927 vs. 849 pg/ml, p<0.05). This indicates that in early onset T2D, PBMC are more sensitive to low doses of the TLR4 activator LPS, as well as the fatty acid palmitate. Thus, it may be that the consumption of even low levels of lipids cause a greater inflammatory reaction for individuals with T2D than for obese individuals without T2D.

PBMC consist of monocytes and lymphocytes. Macrophage/monocyte populations are key producers of pro-inflammatory cytokines (39). Here, monocyte behavior in early onset T2D (n=3) relative to obese controls (n=3) was examined (Fig. 2B). Independent of TLR4 activator, the percentage of monocytes producing TNF-α and IL-1β was greater for the T2D cohort relative to the cohort without T2D. The difference in TNF-α activity observed between the PBMC cultures (24 hour) and the intracellular assays (4 hour) may suggest that initially monocytes are more reactive with respect to TNF-α production but level out with time. Although within a 4-hour culture monocytes are the primary source of LPS- and palmitate-induced TNF-α and IL-1β production (data not shown), the response of other cells within the PBMC may eventually dilute out the initial differences in monocyte activity. The greater reactivity of monocytes for IL-1β synthesis supports the findings with PBMC 24-hour cultures, suggesting that the IL-1β response is more sustained.

### Cytokine activity

This dichotomy in TNF-α and IL-1β activity may reflect physiological differences between obese states relative to T2D. TNF-α has been implicated in the pathology underlying obesity and T2D. Nonetheless, there is inadequate information on PBMC TNF-α production in obesity or the metabolic syndrome in adults. Much less is known in paediatric populations (40). TNF-α-mediated processes may be more involved in the complications associated with T2D such as cardiovascular disease (41,42). Conversely, IL-1β is considered an instigator of metabolic disease due to its capacity to drive sterile inflammation (43). Extensive studies in humans and animal have found that IL-1β, or inflammasome components required for the secretion of IL-1β, are increased in metabolic disease (reviewed in Refs. (44,45). Moreover, treatment with IL-1β antagonists can improve glycaemia in adults with T2D and in animal models of T2D (46,47). Here, IL-1β levels did not correlate with physical parameters or clinical chemistry, but this may be due to the small sample size. The narrow BMI range may also have limited analysis of immune activity with respect to BMI.

### Study limitations

First, the sample size was small raising the likelihood of type 1 or 2 errors in the statistical analysis. This is particularly the case with the intracellular cytokine staining. Second, there were significantly more First Nations individuals in the T2D cohort compared to controls, making it possible that this was an effect based on ethnic differences in immunity. However, when analyzed against ethnicity IL-1β production after exposure to LPS or palmitate did not differ between First Nations and non-First Nations individuals (data not shown). In addition, IL-1β synthesis by PBMC from First Nations with T2D (n=7) was 3- and 4-fold greater than that from First Nations without T2D (n=2) following culture of cells with LPS (0.2 µg/ml) and palmitate, respectively (data not shown). Taken together, this supports the premise that the difference in IL-1β activity is due to the presence of T2D and not due to differences in ethnicity. Finally, the exact relationship between the behavior of peripheral PBMC or monocytes and adipose tissue macrophages remains to be determined.

## Summary

Indigenous people appear to have a greater pro-inflammatory physiology likely reflecting environmental–gene interactions (5,6, 24). Due to the greater incidence of early onset metabolic disease in First Nations and other Indigenous populations, we were interested in determining the immune events associated with early onset T2D. Taken together, these initial findings suggest that certain immunological parameters are common in obese youth independent of T2D. PBMC-induced TNF-α synthesis, for example, did not differ between obese adolescents with and without T2D. However, it appears that in early onset T2D, there is a greater susceptibility to IL-1β synthesis upon exposure to low levels of LPS or the fatty acid palmitate. Thus, there may be no safe amount of individuals with T2D to consume.

We are continuing to evaluate systemic and cellular immunity in early onset T2D, in conjunction with age- and BMI-matched controls. T2D presents a serious burden to the First Nations community. The goal of our studies is an improved understanding of the biology behind T2D in First Nations children in support of new therapeutics in the prevention of T2D and related complications.
